# Delayed Improvement of Left Ventricular Function in Newly Diagnosed Heart Failure Depends on Etiology—A PROLONG-II Substudy

**DOI:** 10.3390/s22052037

**Published:** 2022-03-05

**Authors:** Johanna Mueller-Leisse, Johanna Brunn, Christos Zormpas, Stephan Hohmann, Henrike Aenne Katrin Hillmann, Jörg Eiringhaus, Johann Bauersachs, Christian Veltmann, David Duncker

**Affiliations:** Hannover Heart Rhythm Center, Department of Cardiology and Angiology, Hannover Medical School, Carl-Neuberg-Str. 1, 30625 Hannover, Germany; mueller-leisse.johanna@mh-hannover.de (J.M.-L.); johanna@brunn-online.net (J.B.); zormpas@vinzenzkrankenhaus.de (C.Z.); hohmann.stephan@mh-hannover.de (S.H.); hillmann.henrike@mh-hannover.de (H.A.K.H.); eiringhaus.joerg@mh-hannover.de (J.E.); bauersachs.johann@mh-hannover.de (J.B.); c.veltmann@ep-bremen.com (C.V.)

**Keywords:** HFrEF, heart failure, WCD (wearable cardioverter-defibrillator), ICD (implantable cardioverter-defibrillator), LVEF (left ventricular ejection fraction), SCD (sudden cardiac death), PPCM (peripartum cardiomyopathy)

## Abstract

In patients with newly diagnosed heart failure with reduced ejection fraction (HFrEF), three months of optimal therapy are recommended before considering a primary preventive implantable cardioverter-defibrillator (ICD). It is unclear which patients benefit from a prolonged waiting period under protection of the wearable cardioverter-defibrillator (WCD) to avoid unnecessary ICD implantations. This study included all patients receiving a WCD for newly diagnosed HFrEF (*n* = 353) at our center between 2012 and 2017. Median follow-up was 2.7 years. From baseline until three months, LVEF improved in patients with all peripartum cardiomyopathy (PPCM), myocarditis, dilated cardiomyopathy (DCM), or ischemic cardiomyopathy (ICM). Beyond this time, LVEF improved in PPCM and DCM only (10 ± 8% and 10 ± 12%, respectively), whereas patients with ICM showed no further improvement. The patients with newly diagnosed HFrEF were compared to 29 patients with a distinct WCD indication, which is an explantation of an infected ICD. This latter group had a higher incidence of WCD shocks and poorer overall survival. All-cause mortality should be considered when deciding on WCD prescription. In patients with newly diagnosed HFrEF, the potential for delayed LVEF recovery should be considered when timing ICD implantation, especially in patients with PPCM and DCM.

## 1. Introduction

Patients with newly diagnosed heart failure with reduced ejection fraction (HFrEF) are at risk of sudden cardiac death [1,2,3,4]. In the current guidelines, a primary preventive implantable cardioverter-defibrillator (ICD) is indicated to prevent sudden cardiac death in these patients, if left ventricular ejection fraction (LVEF) remains reduced after three months of optimized heart failure therapy [5]. However, modern heart failure therapy has led to an improved prognosis for HFrEF patients, and a reduction in both sudden and non-sudden deaths [6,7,8]. Meanwhile, complication rates of ICD therapy remain non-negligible [9,10,11]. Despite this, a large percentage of ICD implantations performed are non-indicated [12]. Therefore, thorough risk stratification to avoid unnecessary ICD implantations is crucial [13,14,15,16]. In the PROLONG study, extending the time of therapy optimization beyond three months under the protection of the wearable cardioverter-defibrillator (WCD) to await further LVEF improvement in some patients with newly diagnosed HFrEF has been proposed [13]. However, which patients benefit from such prolonged risk stratification is unclear [3,17]. WCD prescription and the timing of ICD implantation remain controversial, and further data on LVEF recovery potential and long-term prognosis are warranted [14,15,18,19]. Furthermore, the WCD has been criticized for its high cost and no proven survival benefit in a randomized controlled trial [20,21,22]. In the PROLONG-II study, it was shown that overall survival after the WCD was favorable, including in patients after a WCD shock. These results argued against a mere mortality shift from sudden to non-sudden deaths by the WCD [23]. The aim of the present analysis was to evaluate the potential for LVEF improvement, and the incidence of early and late life-threatening arrhythmias and mid-term mortality in different entities of HFrEF. We also compared the outcomes of patients with newly diagnosed HFrEF with patients receiving the WCD after explantation of a previously implanted ICD. Explantation of an infected ICD is another indication for the WCD, which seems less controversial compared to the indication in newly diagnosed HFrEF [24,25,26]. However, proof of an overall long-term survival benefit in this patient group is also lacking, and data on long-term follow-up and prognosis are warranted [27].

## 2. Methods

The PROLONG-II study was an observational single-center study evaluating the prognosis of patients with HFrEF after WCD prescription. Detailed trial design has been described elsewhere [23]. In brief, patients receiving a WCD (LifeVest, Zoll, Pittsburgh, PA, USA) for newly diagnosed HFrEF at Hannover Medical School between 2012 and 2017 were included. WCD prescription time was at minimum three months, but was extended in patients with newly diagnosed HFrEF when further LVEF improvement was anticipated to avoid unnecessary ICD implantations, according to previously described criteria from the PROLONG study. Borderline LVEF value at three months (30–35%) marked an increase in LVEF compared to baseline (≥5%) and nonoptimized heart failure therapy [13].

The present study also analyzed a second, distinct patient cohort consisting of patients receiving the WCD after an explanted ICD during the same time period.

The study complies with the Declaration of Helsinki and was approved by the local ethics committee. All patients gave informed consent.

Data including clinical status and clinical history, medication, basic laboratory parameters, electrocardiograms, left ventricular function in echocardiography, and/or cardiac magnetic resonance imaging and WCD data, including wear time and arrhythmic episodes, were acquired at baseline, at three months, and at last available follow-up, and were derived from inpatient visits, outpatient visits and/or telephone interviews, and from the remote monitoring platform of the manufacturer of the WCD. Renal disease was defined as known renal damage, chronic eGFR < 60 mL/min/1.73 or acute kidney injury according to KDIGO criteria. WCD therapies occurring in the context of unstable fast ventricular tachycardia (>200 beats per minute) or ventricular fibrillation were considered appropriate. Patients alive at the last available follow-up were labelled as censored.

### Statistics

Statistical analysis was performed using SPSS Statistics version 26 (IBM Corp., Armonk, NY, USA). Continuous variables were reported as median and range or mean with standard deviation. Wilcoxon test, Mann–Whitney U test, Kruskal–Walllis test and Friedman’s test were used for associations, as appropriate. Categorical variables were reported as numbers and percentages, and the chi-squared test or binary logistic regression analysis were used for associations. Moreover, *p*-values < 0.05 were considered statistically significant, after adjustment for multiple testing by Bonferroni correction where appropriate. The first and last authors take full responsibility for data integrity and analysis.

## 3. Results

### 3.1. Baseline Characteristics

The PROLONG-II study included 353 patients (69% male; mean age 56 ± 15 years) with newly diagnosed HFrEF (mean LVEF 25 ± 8%). HFrEF etiologies were ischemic cardiomyopathy (ICM) in 126 patients (36%), dilated cardiomyopathy (DCM) in 169 patients (48%), peripartum cardiomyopathy (PPCM) in 27 patients (7%), myocarditis in 24 patients (7%), and other non-ischemic cardiomyopathy including amyloidosis, hypertrophic obstructive cardiomyopathy, and congenital heart disease in seven patients (2%). Baseline characteristics of the patients are presented in Table 1. Patients with PPCM were younger compared to ICM (*p* < 0.001), DCM (*p* < 0.001) and myocarditis (*p* = 0.005); patients with myocarditis were younger than ICM and DCM patients (*p* < 0001); and patients with DCM were younger than patients with ICM (*p* < 0.001). Baseline LVEF was significantly lower in patients with PPCM and DCM compared to ICM (*p* = 0.003 each). Baseline NYHA functional class was highest in patients with PPCM. Differences in NT-proBNP levels were not statistically significant. Patients with ICM had more cardiovascular risk factors, while patients with DCM more often had renal disease.

In addition to the patients with newly diagnosed HFrEF, we also analyzed a second, distinct patient cohort receiving the WCD after explantation of an ICD. This group included 29 patients (86% male; aged 67 ± 13 years). Characteristics of the patients with an explanted ICD compared to patients with newly diagnosed HFrEF are presented in Table 2. Patients with an explanted ICD were significantly older (*p* = 0.036), had higher LVEF values (*p* < 0.001), lower NYHA class (*p* = 0.002) and lower NT-proBNP levels (*p* < 0.001). They had more comorbidities such as diabetes (*p* = 0.026), dyslipidemia (*p* < 0.001) and renal disease (*p* = 0.005). Underlying heart disease of the patients was ICM in 17 (59%), DCM in seven (24%), and other in five (17%). ICD indication was the secondary prevention in 13 patients (45%).

### 3.2. Follow-Up

Follow-up of patients with newly diagnosed HFrEF was 2.8 ± 1.5 years (2.9 ± 1.6 for ICM patients, 2.7 ± 1.5 for DCM, 3.2 ± 1.5 for PPCM, 2.4 ± 1.3 for myocarditis and 2.9 ± 1.6 for others). Twenty patients were lost to follow-up (five with ICM, twelve with DCM, two with myocarditis, and one with cardiac amyloidosis). The mean WCD wear time of patients with newly diagnosed HFrEF was 77 ± 44 for patients without a prolonged WCD prescription and 184 ± 93 days for patients with a prolonged WCD prescription. Mean daily wear time was 22 ± 4 h. There were no significant differences in WCD wear time between HFrEF groups.

In patients with an explanted ICD, the mean follow-up period was 3.2 ± 1.8 years and one patient was lost to follow-up (3%). Patients with an explanted ICD had a mean WCD wear time of 55 ± 45 days and a daily wear time of 22 ± 1 h.

### 3.3. Medication

In patients with newly diagnosed HFrEF, medical heart failure therapy was initiated at the time of first diagnosis and optimized during hospital stay and further follow-ups. Beta-blockers were initiated in 94 %, angiotensin-converting enzyme inhibitors (ACEI) or angiotensin II receptor blockers (ARB) in 96 % and mineralocorticoid receptor antagonists (MRA) in 88 % of patients. Diuretics were prescribed in 81%, ivabradine in 22%, and digitalis in 9% of patients. From the initialization of medical therapy until three months follow-up, drug dosages of beta-blocker, ACEI/ARB and/or MRA were increased in 61% of patients (beta-blocker in 36%, ACEI/ARB in 42%, and MRA in 22%). Between three months follow-up and last follow-up, another increase in dosages was noted in 45% of patients (beta-blocker in 26%, ACEI/ARB in 24%, and MRA in 17%). Medication was not significantly increased during the follow-up period in the patient cohort receiving the WCD after ICD explantation.

### 3.4. LVEF Recovery

**Figure 1:**
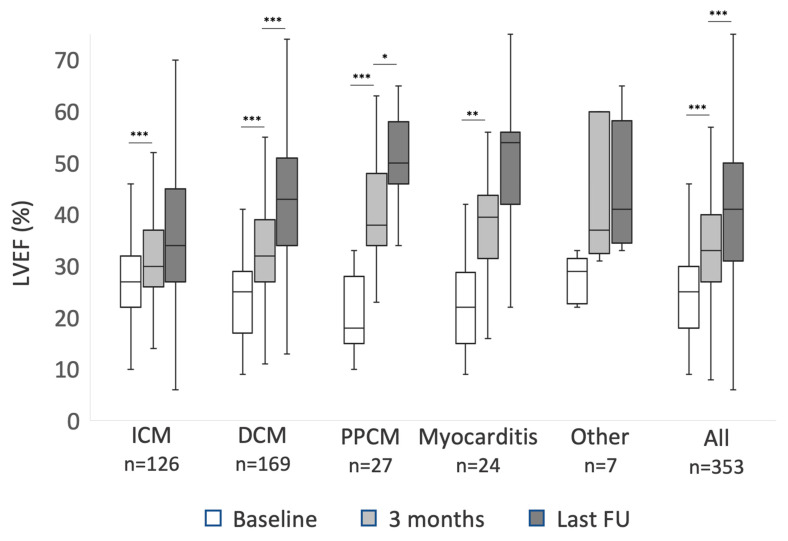
The improvement of left ventricular ejection fraction (LVEF) during follow-up of patients with newly diagnosed heart failure with reduced ejection fraction (HFrEF): mean values at baseline, three months, and last available follow-up (FU) for patients with ischemic cardiomyopathy (ICM), dilated cardiomyopathy (DCM), peripartum cardiomyopathy (PPCM), myocarditis and other diagnosis. Asterisks mark *p*-values < 0.05 (*), <0.01 (**) and <0.001 (***). Difference between 3 months and last FU LVEF in patients with myocarditis did not meet statistical significance with *p* = 0.09.

In patients with newly diagnosed HFrEF, LVEF showed significant improvement within the first three months in all ICM, DCM, PPCM, and myocarditis (*p* < 0.001 each). The degree of LVEF improvement differed. Within the first three months, patients with DCM improved significantly more than patients with ICM (9 ± 9% vs 5 ± 8%, *p* < 0.002). Patients with PPCM improved 20 ± 10%, significantly more compared to both ICM and DCM (*p* < 0.001 each). Patients with myocarditis improved 15 ± 9% (*p* < 0.001 compared to ICM and 0.08 compared to DCM). After three months, LVEF continued to improve significantly until last follow-up in patients with both PPCM (10 ± 8%; *p* < 0.024) and DCM (10 ± 12%; *p* < 0.001), but not significantly in patients with ICM, myocarditis, or other diagnoses. Data on LVEF recovery are summarized in Figure 1. NYHA functional class also improved in all subgroups within the first three months (*p* < 0.001 each), but beyond three months NYHA functional class improved in PPCM (*p* = 0.03) and DCM (*p* = 0.02) only. In the patient cohort with an explanted ICD, neither LVEF nor NYHA class changed significantly during follow-up.

### 3.5. ICD Implantations

**Figure 2:**
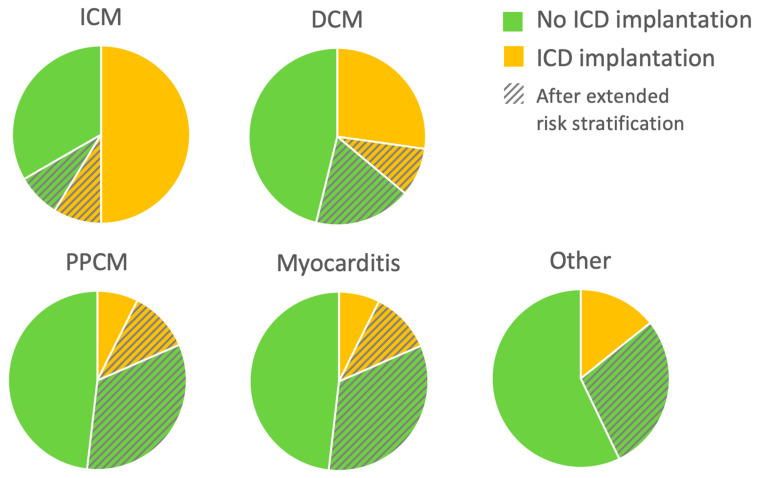
Implantable cardioverter-defibrillator (ICD) implantations during follow-up (FU). For each etiology of heart failure with reduced ejection fraction (HFrEF), proportion of patients meeting ICD implantation criteria or not after the wearable cardioverter-defibrillator (WCD) are depicted. Pie charts also show the proportion of patients with an extended period of therapy optimization before decision-making. n = 353: 126 ICM (ischemic cardiomyopathy), 169 DCM (dilated cardiomyopathy), 27 PPCM (peripartum cardiomyopathy), 24 myocarditis, and seven other NICM.

Patients with newly diagnosed HFrEF who experienced appropriate WCD shocks while wearing WCD received an ICD for secondary prevention. A primary preventive ICD was implanted after three months only in cases where no further LVEF recovery was anticipated, based on previously described criteria [13]. These ICD implantation criteria were met in 63 patients with ICM (50%), 46 patients with DCM (27%), 2 patients with PPCM (7%), 1 with myocarditis (4%), and one with congenital heart disease. Eighty-eight patients (25%) still had an LVEF ≤ 35% at three months, but further LVEF improvement was anticipated. The risk stratification period under protection of the WCD was prolonged in this group of patients. Twenty-one of them had ICM (17%), 45 had DCM (27%), 12 had PPCM (44%), 8 had myocarditis (33%), and 2 had other diagnoses (one with cardiac involvement of eosinophilic granulomatosis with polyangiitis and one with cardiac transplant vasculopathy). Of these patients with prolonged WCD wearing, 31 (35%) received an ICD after the extended WCD period: 11 with ICM (52%), 15 with DCM (33%), 3 with PPCM (25%) and 2 with myocarditis (25%). These data are illustrated in Figure 2.

### 3.6. Ventricular Arrhythmias

To assess the risk of early and late sudden cardiac death in newly diagnosed HFrEF, the occurrence of life-threatening ventricular arrhythmias (fast ventricular tachycardia > 200 beats per minute or ventricular fibrillation) treated by the WCD or a later implanted ICD was assessed. Patients without an ICD recommendation after the WCD did not experience clinically relevant ventricular arrhythmias or sudden cardiac death during follow-up.

Patients with PPCM were most likely to experience early life-threatening arrhythmias treated by the WCD (11%), but had no further arrhythmias during long-term follow-up. Patients with ICM and DCM received early WCD shocks in 4% each, and later ICD therapies in 6% and 2%, respectively (16% and 11% of the patients receiving an ICD, respectively; the difference was non-significant). WCD shocks were predictive for appropriate ICD shocks in ICM (*p* = 0.01) and DCM (*p* = 0.02). Patients with myocarditis and other diagnoses had no ventricular arrhythmias.

Among the second, distinct patient group with an explanted ICD, WCD shocks occurred in three patients (10%). During follow-up, a new ICD was implanted in 24 patients (83%), and six of these patients (25%) experienced ICD therapies during follow-up. Compared to the patient cohort with newly diagnosed HFrEF, ICD shocks occurred more often in this explant cohort (*p* = 0.015).

### 3.7. Mortality

**Figure 3:**
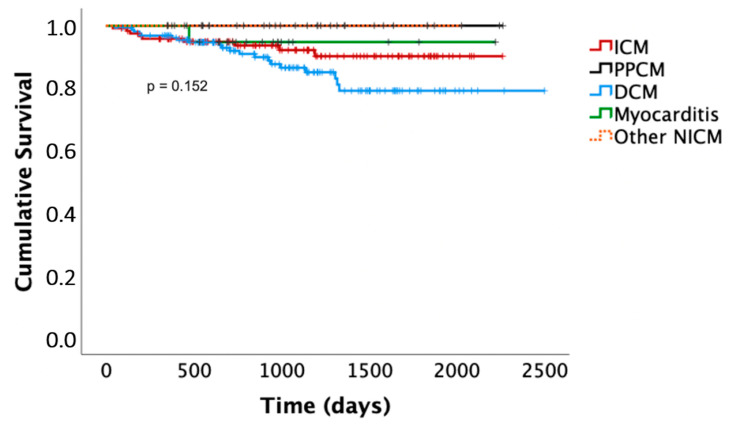
Kaplan–Meier survival curves. ICM, ischemic cardiomyopathy; DCM, dilated cardiomyopathy; PPCM, peripartum cardiomyopathy; NICM, non-ischemic cardiomyopathy.

Death occurred in nine patients with ICM (7%) after 1 ± 1.1 years (one sudden cardiac death in a patient who had refused primary preventive ICD implantation after the WCD, five non-cardiac deaths, three deaths with unknown cause). Death occurred in 20 patients with DCM (12%) after 1.5 ± 1.1 years (4 non-sudden cardiac deaths, 11 non-cardiac deaths, 3 deaths with unknown cause). Death occurred in none of the patients with PPCM and in one patient with myocarditis (4%), who died of relapse myocarditis and septic shock under immunosuppression after 1.1 years. Out of the deaths of unknown cause, none occurred in any of the patients with a prolonged WCD/risk stratification period. Differences in the Kaplan–Meier survival curves did not meet statistical significance. The latter are presented in Figure 3.

**Figure 4:**
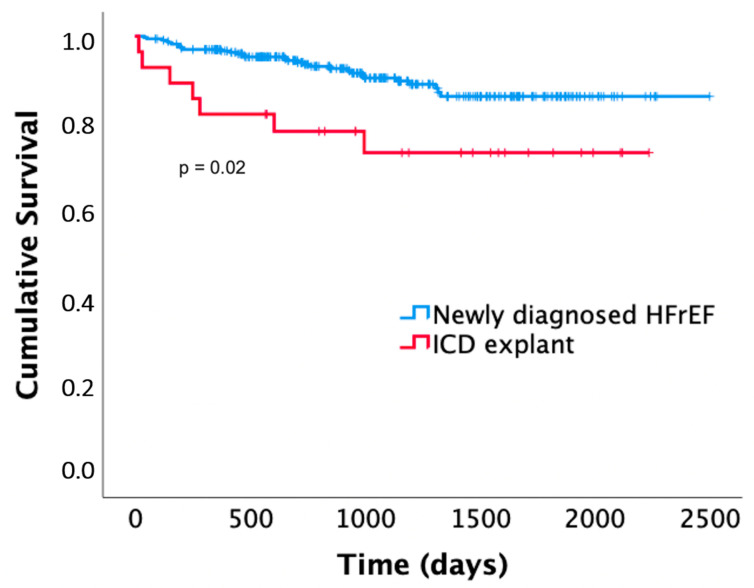
Kaplan–Meier survival curves of patients receiving the WCD for newly diagnosed heart failure with reduced ejection fraction (HFrEF) compared to patients receiving the WCD after ICD explantation.

Patients with an explanted ICD showed significantly higher mortality compared to patients with newly diagnosed HFrEF (*p* = 0.02). Seven (24%) of the patients died after 0.9 ± 1 years (one of sudden cardiac death, one of non-sudden cardiac death, three of non-cardiac death, two with unknown cause of death). These included two out of three patients who had received appropriate WCD shocks during WCD wearing. Death occurred after 0.4 and 0.7 years (the association between WCD shocks and mortality in this patient group was significant; *p* = 0.045). The Kaplan–Meier curves are presented in Figure 4.

## 4. Discussion

While the prognosis of patients with HFrEF is improving, ICD complication rates remain non-negligible [6,7,9,28]. The net benefit of a primary preventive ICD has been questioned by recent trials, especially in patients with non-ischemic cardiomyopathy [29,30,31,32]. As a consequence, guideline recommendations by the ESC were downgraded for patients with non-ischemic cardiomyopathy [5]. We and others have previously argued that a prolonged time should be taken for therapy optimization and risk stratification before deciding on ICD implantation [13,14,15]. The PROLONG and PROLONG-II study have suggested that ICD implantations can be avoided in some patients with newly diagnosed HFrEF by this practice without increasing the risk of sudden cardiac death [13,23]. The present analysis was conducted to evaluate which patients benefit from an extended waiting period. The main findings were:

1. In patients with newly diagnosed HFrEF, LVEF improved significantly beyond the first three months of optimized heart failure therapy in patients with DCM and PPCM;
2. Following the PROLONG protocol, a minority of these patients met criteria for ICD implantation;
3. Patients receiving the WCD because of prior ICD explantation showed a higher all-cause mortality compared to patients with newly diagnosed HFrEF.

### 4.1. Baseline Characteristics

Baseline characteristics of patients with HFrEF were similar compared to other heart failure studies [29,31,33,34,35]. As expected, there were age and gender differences between HFrEF entities and also differences in terms of comorbidities, which play an important role in the prognosis of heart failure patients [36,37,38,39,40,41]. As expected, patients with PPCM were younger and had few comorbidities [42,43]. As was also observed in other studies, patients with DCM were younger than patients with ICM, had fewer comorbidities, and a lower baseline LVEF [44,45]. Patients with DCM in our cohort more often had renal disease, indicating a sicker patient population. Patients with myocarditis were also sicker and older compared to most myocarditis trials [46,47,48,49,50]. However, these trials included patients with mildly reduced and preserved LVEF, while patients with myocarditis included in the present study had exclusively reduced ejection fraction.

The present study also analyzed a second, distinct patient cohort receiving the WCD after ICD explantation due to infection. These patients were older compared to patients with newly diagnosed HFrEF. Patients with ICD explantation typically have long-standing chronic heart failure and optimized medical therapy is established. Therefore, not surprisingly, these patients had lower NT-proBNP levels, lower NYHA class, higher LVEF values, and several comorbidities. Ischemic cardiomyopathy was the most common underlying heart disease, around half of the patients had a secondary preventive ICD indication, and they were predominantly male. Similar characteristics have also been described by others [51,52,53,54,55].

### 4.2. LVEF Recovery under Optimized Therapy

The guideline-directed medical therapy that was initiated after the diagnosis of HFrEF was more guideline-accordant than in most other heart failure studies and real-world data, with beta-blockers prescribed in 94%, ACEI/ARB in 96%, and MRA in 88% of patients [29,56,57,58]. This is likely related to the fact that the study was conducted at a single specialized heart failure center. Heart failure medication was further increased in 60% of patients within the first three months following diagnosis, and in 45% beyond that time. WCD compliance was excellent in the PROLONG-II study. To improve patient adherence, patients were contacted when WCD wear-time alerts occurred [23]. Compliance is crucial for effectiveness of the WCD. In contrast to the present study, WCD compliance was poor in the randomized controlled VEST trial, which failed to show a survival benefit of the WCD in the intention-to-treat analysis [20,59]. These data illustrate the advantages of referring heart failure patients to a heart failure center or heart failure specialist. The importance of close patient follow-up and education was also demonstrated by others [60,61,62]. Moreover, three months may not be enough time to establish individual optimized medication in many patients. Of note, the present study was initiated in 2012, and therefore before the approval and recommendation of angiotensin receptor-neprilysin inhibitors (ARNI) and SGLT-2 inhibitors. According to current guidelines, all beta-blockers, ACEI/ARB or ARNI, MRA and SGLT-2 should be initiated after diagnosis [5]. As HFrEF therapy becomes more complex, the establishment of stable optimized therapy may take even more time [14,58].

As expected after our previous data analysis, LVEF improved significantly under treatment [13]. The present study shows significant differences in LVEF recovery among HFrEF entities. Patients with PPCM and myocarditis showed the most marked LVEF recovery, and patients with ICM the least. Patients with PPCM and DCM showed another improvement in LVEF beyond the first three months, while patients with ICM and myocarditis did not. This finding is in line with the overall expected good prognosis of patients with PPCM, but importantly, a similar further LVEF improvement of around 10% was observed in DCM patients. Patients with ICM may have reduced potential for reverse remodeling due to scar formation [63,64,65]. In myocarditis, further improvement may have been expected [66], and was seen as a trend in the present study. Mean LVEF values suggested a relevant improvement beyond three months, but statistical significance was missed, most likely due to the small sample size. Moreover, patients with myocarditis represent a very heterogenous group with heterogenous response to treatment [67,68,69].

In patients with an explanted ICD, neither a relevant escalation of heart failure therapy nor improvement of LVEF was noted, confirming the fact that these patients had chronic heart failure under already stable medical therapy. In contrast to patients with newly diagnosed heart failure, patients with chronic heart failure have lower overall potential for clinical improvement [70,71,72,73,74].

### 4.3. Ventricular Arrhythmias and Implications for ICD Timing

To elucidate the benefit of a WCD and/or ICD, the incidences of early and late life-threatening ventricular arrhythmias were studied. It has been shown previously that patients with both ICM and NICM experience life-threatening arrhythmias following diagnosis [1,2,3,4]. In the present study, patients with PPCM experienced early, but no late arrhythmias: 11% received WCD shocks, but no later ICD shocks occurred. This is in accordance with the good long-term prognosis of patients with PPCM despite the early risk of arrhythmias, supporting the use of the WCD and questioning ICD primary preventive therapy in this particular patient group [42,75,76]. The young age of this patient group must also be considered in terms of risk for long-term complications associated with ICD therapy [77,78]. In patients with ICM and DCM, on the other hand, both early and late ventricular arrhythmias occurred, and WCD shocks were predictive for ICD shocks. Patients without an ICD recommendation after the PROLONG protocol did not suffer from ventricular arrhythmias or sudden cardiac death during extended follow-up, which was an important confirmatory finding concerning the safety of the prolonged risk stratification with less ICD implantations [13,23].

Patients with an explanted ICD experienced WCD shocks in 10% of cases and were also likely to receive ICD therapies after re-implantation (25% of patients). These data confirm the high arrhythmic risk in these patients [24,25,27,79].

### 4.4. Overall Mortality

We have previously shown that the overall prognosis of patients with newly diagnosed HFrEF wearing the WCD during thorough therapy optimization is favorable compared to historical cohorts, and that ICD implantations can be avoided without signs of an increased risk of sudden cardiac death [13,23]. Analysis of the different HFrEF entities in the present study shows that prognosis is especially favorable in patients with PPCM. Only four patients received an ICD (15%). None of the patients died after a mean follow-up of 3.2 ± 1.5 years. Differences in overall mortality between the other diagnoses were not statistically significant, although Kaplan–Meier curves suggested a worse survival in DCM patients. Other studies have suggested that DCM patients have an overall slightly better survival compared to ICM patients [80]. However, DCM patients in the present study often had renal disease, which is an important prognostic marker and may partly explain the poorer survival of DCM patients in our study [44].

Patients with a previously explanted ICD showed poorer survival compared to patients with newly diagnosed heart failure. Besides suffering from chronic heart failure and comorbidities, ICD infection and explantation procedure also confer a high mortality risk [81,82].

### 4.5. Implications for ICD Timing and WCD Prescription

Optimal timing of ICD evaluation may be challenging in patients with HFrEF, as patients have a risk of sudden cardiac death, but their long-term risk is unknown at the time of diagnosis [14,15]. Most studies investigating ICD therapy were conducted before the era of modern pharmacological heart failure therapy [83,84,85,86,87]. Since then, the prognosis of heart failure patients has improved [11,40]. Because of relevant complication rates, unnecessary ICD implantations should be avoided and ICD indications evaluated critically [9,88]. In fact, ICD implantation numbers have recently declined [89,90]. The present study suggests that patients with DCM and especially PPCM have the potential for continuing LVEF recovery under continued optimized treatment, resulting in less ICD indications. Patients with ICM, on the other hand, showed less reverse remodeling, which is in concordance with previous observations [63,65]. Most patients with ICM did receive an ICD despite prolonged risk stratification, and the patients did not show significant recovery beyond the first three months. Therefore, HFrEF entity should be considered when evaluating ICD indication.

We performed the extended risk stratification under temporary protection of the WCD. The present study shows a relevant incidence of WCD shocks, supporting the use of the WCD during risk stratification in newly diagnosed HFrEF. According to our and other data, patients with PPCM are particularly suited for prolonged WCD therapy and WCD therapy in general, as they show relevant early ventricular arrhythmias but have an overall good prognosis, and rarely seem to need long-term ICD therapy [75,91]. These observations might also be valid for young patients with non-ischemic cardiomyopathy without comorbidities in general. Concerning the costs of a prolonged WCD therapy, these should also be weighed against the costs of life-long ICD therapy [92,93,94].

Another WCD indication is the explantation of a previously implanted ICD, which seems to be less controversially discussed in comparison to the indication in newly diagnosed HFrEF [24,53,95,96,97]. However, there is a lack of evidence of a survival benefit of the WCD in this patient group, and a paucity of data on prognosis and arrhythmia burden. Our study results show a relevant number of arrhythmias in this patient group. However, overall mortality is also high. It remains unclear whether the WCD conveys a long-term survival benefit in this population [27].

## 5. Conclusions

In newly diagnosed HFrEF, underlying heart disease should be considered when deciding on the timing of ICD implantation. An extended risk stratification under optimized treatment and the temporary protection of the WCD may be considered, particularly in patients with DCM, PPCM, and possibly myocarditis, who show marked LVEF recovery beyond the first three months after diagnosis.

## 6. Limitations

PROLONG-II was a retrospective observational study conducted at a specialized heart failure center experienced with WCD therapy. Thus, patient adherence to therapy was high. To improve compliance and optimization of therapy, patients were contacted frequently. While this may limit generalizability, it was the aim of the study to show LVEF recovery under optimized, guideline-directed heart failure therapy. Notably, treatment with newer heart failure drugs was not assessed in the study. The group of patients with myocarditis was small and heterogenous, with no ventricular arrhythmias during follow-up. Therefore, limited conclusions can be drawn for this specific entity.

## Figures and Tables

**Table 1:** Baseline characteristics of different HFrEF etiologies.

	All HFrEF	ICM	DCM	PPCM	Myocarditis	Other	*p*-Value
Patients, n (%)	353 (100)	126 (35)	169 (48)	27 (7)	24 (7)	7 (2)	
Male, n (%)	244 (69)	107 (85)	118 (70)	0 (0)	15 (63)	4 (57)	<0.001
Age, years	56 ± 15	64 ± 11	56 ± 14	34 ± 4	50 ± 14	47 ± 17	<0.001
LVEF, %	25 ± 8	27 ± 8	24 ± 7	21 ± 7	23 ± 9	28 ± 4	<0.001
NYHA functional class	2.7 ± 0.7	2.7 ± 0.7	2.6 ± 0.7	3.2 ± 0.7	2.8 ± 0.7	2.3 ± 0.5	0.008
NT-proBNP, ng/L	6549 ± 8565	5197 ± 6847	7959 ± 10,017	5713 ± 7621	4571 ± 2865	2455 ± 2418	0.388
Heart rate, bpm	82 ± 23	80 ± 19	82 ± 20	85 ± 19	89 ± 45	77 ± 20	0.646
AF, n (%)	58 (17)	21 (17)	33 (20)	1 (4)	3 (13)	0 (0)	0.196
QRS duration, ms	116 ± 29	114 ± 27	118 ± 31	104 ± 28	112 ± 22	118 ± 23	0.05
LBBB, n (%)	65 (19)	17 (14)	37 (22)	6 (22)	4 (17)	1 (14)	0.706
Pacemaker, n (%)	10 (3)	6 (5)	4 (2)	0 (0)	0 (0)	0 (0)	0.474
Hypertension, n (%)	196 (56)	98 (78)	81 (48)	5 (19)	10 (42)	2 (29)	<0.001
Diabetes, n (%)	81 (23)	54 (43)	20 (12)	2 (7)	4 (17)	1 (14)	<0.001
Family history of CVD, n (%)	57 (16)	26 (21)	23 (14)	4 (15)	4 (17)	0 (0)	0.397
Dyslipidemia, n (%)	119 (34)	77 (61))	37 (22)	1 (4)	4 (17)	0 (0)	<0.001
Smoking, n (%)	157 (45)	67 (53)	72 (43)	5 (19)	12 (50)	1 (14)	0.006
Alcohol abuse, n (%)	6 (2)	2 (2)	4 (2)	0 (0)	0 (0)	0 (0)	0.833
Renal disease, n (%)	77 (22)	24 (19)	46 (27)	1 (4)	4 (17)	2 (29)	0.056

HFrEF, heart failure with reduced ejection fraction; ICM, ischemic cardiomyopathy; DCM, dilated cardiomyopathy; PPCM, peripartum cardiomyopathy; LVEF, left ventricular ejection fraction; NYHA, New York Heart Association; AF, atrial fibrillation; LBBB, left bundle branch block; CVD, cardiovascular disease.

**Table 2:** Baseline characteristics of patients with an explanted ICD versus patients with newly diagnosed HFrEF (PROLONG-II cohort).

	Explant Cohort	PROLONG-II Cohort	*p*-Value
Patients, n	29	353	
Underlying heart disease			<0.001
ICM	17 (59)	126 (35)	
DCM	7 (24)	169 (48)	
PPCM	0	27 (7)	
Myocarditis	0	24 (7)	
Other	5 (17)	7 (2)	
Male, n (%)	25 (86)	244 (69)	0.036
Age, years	67 ± 13	56 ± 15	<0.001
LVEF, %	34 ± 13	25 ± 8	<0.001
NYHA functional class	2.2 ± 0.8	2.7 ± 0.7	0.002
NT-proBNP, ng/L	2743 ± 5462	6549 ± 8565	<0.001
Heart rate, bpm	77 ± 17	82 ± 23	0.207
AF, n (%)	3 (10)	58 (17)	0.421
QRS duration, ms	132 ± 33	116 ± 29	0.025
LBBB, n (%)	4 (14)	65 (19)	0.787
Hypertension, n (%)	19 (66)	196 (56)	0.297
Diabetes, n (%)	12 (41)	81 (23)	0.026
Family history of CVD, n (%)	5 (17)	57 (16)	0.878
Dyslipidemia, n (%)	19 (66)	119 (34)	0.001
Smoking, n (%)	15 (52)	157 (45)	0.451
Alcohol abuse, n (%)	1 (3)	6 (2)	0.5
Renal disease, n (%)	13 (45)	77 (22)	0.005

ICM, ischemic cardiomyopathy; DCM, dilated cardiomyopathy; PPCM, peripartum cardiomyopathy; LVEF, left ventricular ejection fraction; NYHA, New York Heart Association; AF, atrial fibrillation; LBBB, left bundle branch block; CVD, cardiovascular disease.

## Data Availability

Raw data were generated at Hannover Medical School. Derived data are available from the corresponding author upon reasonable request.
